# Will we miss specific IgA detection for the diagnosis of congenital toxoplasmosis? A French retrospective monocenter study on 483 infants

**DOI:** 10.1128/jcm.00379-25

**Published:** 2025-08-11

**Authors:** Lya Hamet, Hélène Guegan, Sorya Belaz, Jean-Pierre Gangneux, Florence Robert-Gangneux

**Affiliations:** 1 Laboratoire de Parasitologie et Mycologie, Centre Hospitalier Universitaire de Rennes, Rennes, France; 2 Univ Rennes, CHU Rennes, Inserm, EHESP, Irset (Institut de Recherche en Santé Environnement Travail) 236344 Rennes, France; Mayo Clinic Minnesota, Rochester, Minnesota, USA

**Keywords:** congenital toxoplasmosis, *Toxoplasma gondii*, serology, IgA, neonatal diagnosis

## Abstract

To estimate the performance of anti-*Toxoplasma* IgA assay for the diagnosis of congenital toxoplasmosis, a retrospective monocenter study was conducted comparing serological results obtained in the framework of routine diagnosis workup. All infants born to mothers infected with *Toxoplasma gondii* during pregnancy from 2010 to 2023 with at least 6 months of serological follow-up were included. Four hundred and eighty-three cases (1,171 sera) were included, of which 56 infants (11.6%) were infected. Twenty out of 60 infants (33.3%) with positive IgA were not infected. The sensitivity to detect IgA antibodies in infected neonates was 71.4%. The specificity was 97.3%. The mean time to detect IgA was 7.7 ± 13.0 days in infected neonates. Anti-*Toxoplasma* IgA was the earliest positive serological test in only two cases (5.0%) but turned negative at 1 month in the absence of specific treatment, suggesting non-specific detection. IgA was associated with anti-*Toxoplasma* IgM, neosynthetized IgG or IgM on comparative Western blotting (WB), or both IgM and neosynthetized IgG or IgM on WB, in 12 cases (30.0%), 2 cases (5.0%), and 19 cases (47.5%), respectively. Sixteen infants with no IgA after birth were diagnosed via neosynthetized IgG or IgM on comparative WB and/or IgM (*n* = 10), or via PCR on amniotic fluid (*n* = 5), or persistent IgG (*n* = 1). Our study suggests that anti-*Toxoplasma* IgA is not a critical serological parameter for the diagnosis of congenital toxoplasmosis, and the recent withdrawal of commercialized reference anti-*Toxoplasma* IgA assays should not affect patient care.

**IMPORTANCE:**

This study will help clinical microbiologists estimate the impact of the withdrawal of anti-*Toxoplasma* IgA reference assays for the diagnosis of congenital toxoplasmosis and will contribute to actualize the recommendations for laboratory diagnosis.

## INTRODUCTION

Toxoplasmosis is a cosmopolitan zoonotic infection caused by an intracellular protozoan, *Toxoplasma gondii*, whose prevalence varies around the world (1). The infection occurs through ingestion of oocysts present in water, food, or soil, or cysts in undercooked infected meat. It can also be acquired through transplantation of an infected organ to a naïve donor or by transfusion. Primary infection with *Toxoplasma gondii* is usually asymptomatic or mildly symptomatic in healthy individuals, except when patients get infected with a virulent genotype strain (1). By contrast, toxoplasmosis is a life-threatening infection in immunocompromised patients, such as HIV-infected patients or transplant patients. Besides, when toxoplasmosis is acquired during pregnancy, the parasite may pass through the placenta and lead to congenital toxoplasmosis with a variable outcome, depending on the stage of pregnancy. Usually, the severity of infection decreases and the rate of transmission increases with gestational age (1, 2). Thus, the fetus can have a wide range of clinical manifestations, with severe neurological damage, such as ventricular dilatations and hydrocephaly, or retinochoroiditis of variable severity, or he can be asymptomatic at birth (3, 4). However, clinical signs can develop after birth, and infected neonates should receive anti-*Toxoplasma* treatment to minimize further sequelae.

In France since 1992, pregnant women benefit from monthly serological monitoring if they are seronegative, as well as information on hygienic and dietary measures to avoid infection (5). The objective is to detect and treat maternal infection as soon as possible, to prevent fetal infection and, if it occurs, decrease the severity of congenital disease (6).

In France, prenatal diagnosis of congenital toxoplasmosis is proposed to women who experienced *Toxoplasma* infection or with strong suspicion of recent infection documented by serological findings (IgG seroconversion or positive IgM associated with low-avidity IgG), with or without ultrasound abnormalities. It relies on *Toxoplasma* DNA detection in amniotic fluid sampled after 18 weeks of gestation and at least 4 weeks after the estimated date of infection. However, a negative result cannot rule out congenital infection, as the sensitivity of prenatal diagnosis is around 90%. Besides, it is rarely done when maternal infection occurs beyond 35 weeks of gestation, at a stage of pregnancy with high transmission risk. For this reason, post-natal screening of newborns is necessary to confirm or exclude congenital toxoplasmosis and guide newborn treatment. The biological tools used for decades include *Toxoplasma* PCR on placenta, cord blood, and more recently on amniotic fluid at birth (7, 8), and serological follow-up of the newborn based on the monitoring of specific IgG, IgM, and IgA antibodies (9). The presence of IgG and IgM neosynthesized by the infant can be confirmed by comparative Western blotting (WB), showing a different pattern from that of the mother (10).

However, the arsenal of commercialized assays is declining, as the Toxo ISAGA assay (BioMérieux, Marcy-l’Etoile, France), long considered the gold standard for the detection of IgM in congenitally infected newborns, was progressively discontinued during 2023, and we showed that Platelia Toxo IgM (Bio-Rad, Marnes-la-Coquette, France) had equivalent performance as Toxo-ISAGA and could replace it (11). During 2024, both Toxo-ISAGA IgA (BioMérieux) and Platelia Toxo IgA (Bio-Rad) have been successively withdrawn from the market. To our knowledge, no other commercialized assay certified for *in vitro* diagnosis remains available for anti-*Toxoplasma* IgA detection in France, meaning that we have lost a tool in the diagnostic arsenal. The aim of this study was therefore to determine if IgA detection in newborns is still of great interest for the diagnosis of congenital toxoplasmosis compared to other serological parameters available, in a country where maternal careful follow-up and treatment are provided.

## MATERIALS AND METHODS

### Study population and samples

This is a retrospective monocentric study at Rennes University Hospital (Rennes, France), aiming at assessing the clinical performance of anti-*Toxoplasma* IgA in the routine post-natal diagnosis of congenital toxoplasmosis. All infants born from mothers infected with *T. gondii* during pregnancy from 2010 to 2023 were included if they benefited from serological follow-up during 6–12 months, showing a clearance of maternal IgG on two consecutive samples.

Prenatal diagnosis of congenital toxoplasmosis was performed by detecting parasitic DNA in the amniotic fluid, 4–5 weeks after the estimated date of infection and after 18 weeks of amenorrhea, using an accredited method already described (8). At birth, newborns benefited from clinical, neurological, and ophthalmological examination (trans-fontanellar ultrasound and ocular fundus), and placentas were tested for *Toxoplasma* by PCR at delivery. The following serological tests were performed: detection of anti-*Toxoplasma* IgG, IgM, and IgA antibodies in cord blood and/or peripheral blood, and comparison of the mother and infant IgG and IgM patterns by WB (WB *Toxoplasma* IgG/IgM; LDBio, Lyon, France) as already described (12). All serological techniques were combined during the first 3 months of life, then only IgG and IgM were monitored for at least 6 months until clearance of maternal IgG on two consecutive samples.

Congenital toxoplasmosis was defined by the following biological criteria: (i) the presence of specific anti-*Toxoplasma* IgM and/or IgA more than 3 days after birth, confirmed by a second sample at least 10 days later; (ii) the presence of stable or increasing IgG titers during follow-up; or (iii) positive *Toxoplasma* PCR on amniotic fluid.

For each neonate, the following information was collected: date of birth, date of serum sampling, IgA and IgM results, outcome (infected and non-infected), trimester of maternal infection, results of comparative Western blotting, and result of *Toxoplasma*-specific PCR on placenta and amniotic fluid when performed. All samples analyzed from infants in the framework of congenital toxoplasmosis diagnosis during 2010-2023 were included if at least one anti-*Toxoplasma* IgA dosage was performed.

### Detection of anti-*Toxoplasma* IgA

Anti-*Toxoplasma* IgA was detected by using either the Toxo-ISAGA IgA assay or the Platelia Toxo IgA. Indeed, in some instances, the delivery of Toxo-ISAGA IgA was discontinued due to manufacturing issues. In that occurrence, the Platelia Toxo IgA assay was used.

The Toxo-ISAGA method is a manual and semiquantitative assay, based on immuno-agglutination of whole tachyzoites of *T. gondii*. According to the manufacturer’s instruction, newborn serum was diluted to 1/20 and incubated in three anti-IgA coated wells for 2 hours at 37°C. After several washing steps, *Toxoplasma* antigen was added and the wells were covered and incubated overnight at 37°C. The agglutination of tachyzoites was observed and rated from 0 (total sedimentation) to 4 (total agglutination) by comparison to negative and positive controls, and the scores obtained for the three wells were summed up. The threshold of positivity is ≥6, while a score of <3 is considered negative.

The Platelia Toxo IgA is a quantitative immunocapture enzyme-linked immunosorbent assay (ELISA) using 96-well microplates coated with anti-human α-chain antibodies, and the reaction steps were automated using an Evolis device (Bio-Rad) following the manufacturer’s protocol. Results were expressed as an index (ratio relative to calibrators). An index of <0.8 is considered negative, while an index of ≥1.00 is considered positive for anti-*Toxoplasma* IgA. Values from 0.8 to 0.99 are considered equivocal (gray zone).

### Statistical methods

The sensitivity of anti-*Toxoplasma* IgA detection for the diagnosis of CT was calculated using the first positive sample obtained from infected babies. Subsequent samples were not included, as treatment is known to decrease antibody synthesis (12); thus, further negative results are the rule. The specificity was calculated using all included sera. The mean time to positive IgA result was calculated for the infants diagnosed with CT and compared with other serological parameters.

For sensitivity and specificity calculation, all results in the gray zone, i.e., Toxo-ISAGA IgA >3 and ≤5 and Platelia Toxo IgA ≥0.8 and <1.0, were considered negative.

## RESULTS

### Included sera

**Fig 1:**
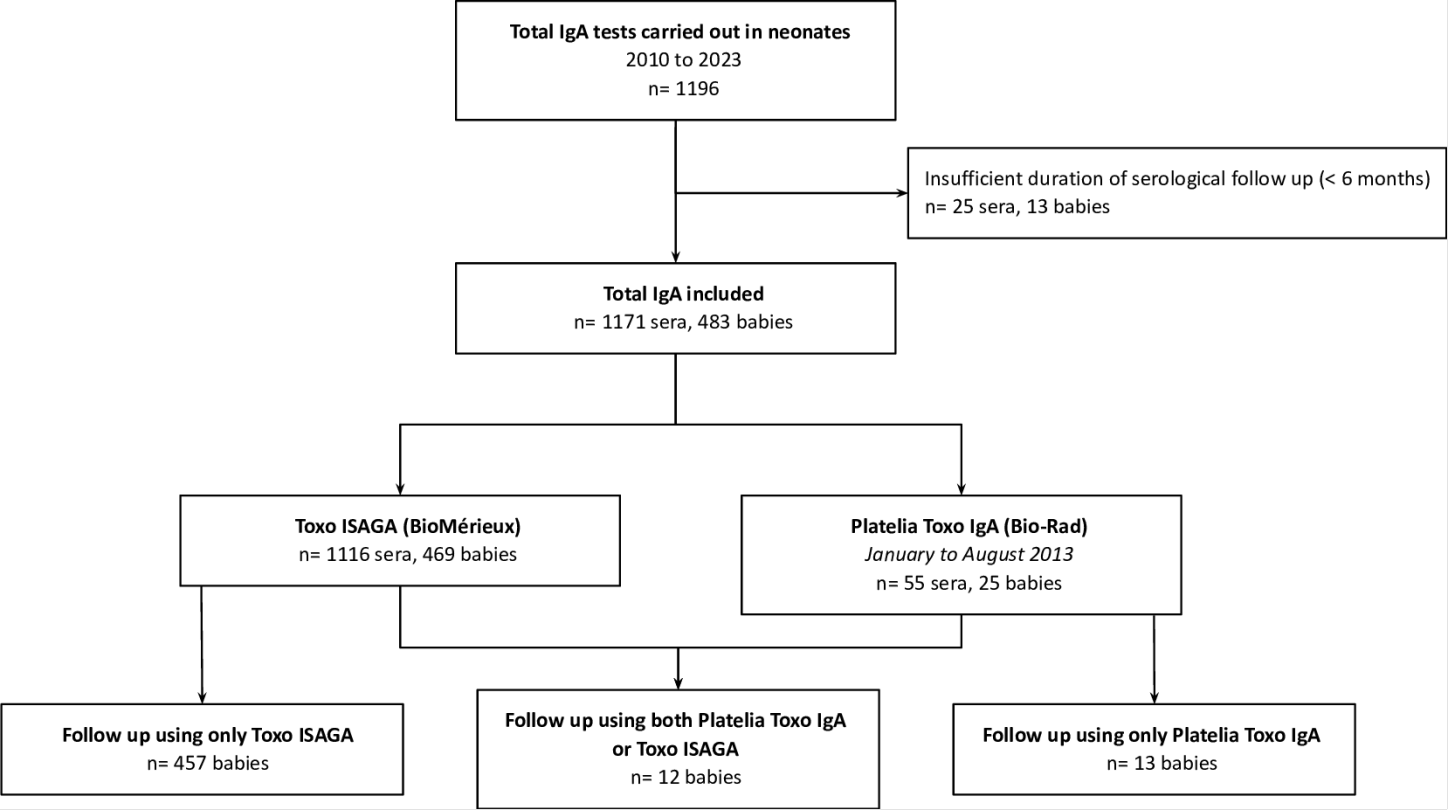
Flowchart of included cases for the analysis of anti-*Toxoplasma* IgA.

A total of 1,196 sera were tested for anti-*Toxoplasma* IgA during 2010–2023, of which 1,171 from 483 infants were included for analysis (Fig. 1). All samples collected at birth and during the follow-up were included; 1,116 (469 neonates) were analyzed using the Toxo-ISAGA IgA assay, and 55 samples (25 neonates) were analyzed by the Platelia Toxo IgA, because of a manufacturer’s shortage of the Toxo ISAGA IgA kit (January 2013–August 2013). Twelve infants were tested alternatively using Toxo-ISAGA IgA or Platelia Toxo IgA (Fig. 1). No discrepancies (ISAGA+/Platelia− or vice-versa) were observed during follow-up. Each neonate had an average of 2.4 sera with IgA detection during follow-up. Of the 483 included babies, 56 (11.6 %) were diagnosed with congenital toxoplasmosis.

### Performance of anti-*Toxoplasma* IgA for the diagnosis of congenital toxoplasmosis

**Fig 2:**
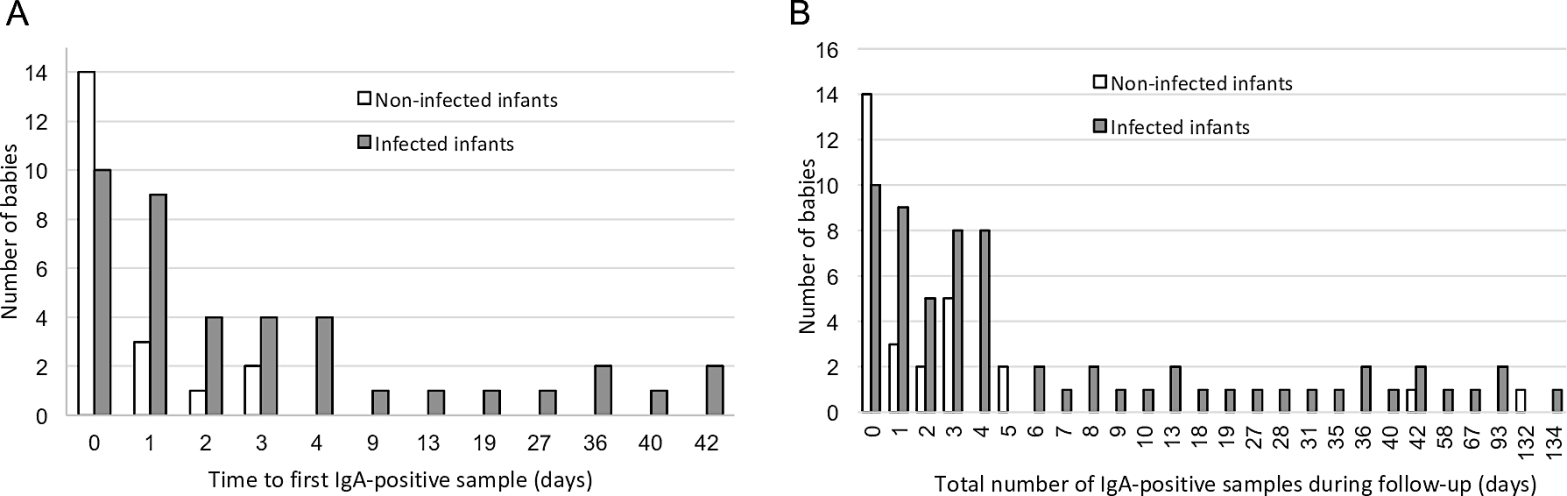
IgA detection according to age of infants with a confirmed (*n* = 40) or excluded (*n* = 20) diagnosis of congenital toxoplasmosis. Age of infants at first detection of positive IgA (**A**). Age of infants at detection of positive IgA (cumulative results) (**B**).

Sixty babies out of 483 (12.4%) had at least one serum with positive IgA during follow-up, corresponding to 93 sera with ISAGA of ≥6, or Platelia of ≥ 1. Of these 60 infants with positive IgA, 40 (66.7%) were congenitally infected, whereas 20 were uninfected. Those 20 uninfected infants had a first positive IgA result exclusively on day 0, day 1, day 2, or day 3 (D3) (mean index rate ± SD = 9 ± 2 for ISAGA, 2.790 for Platelia) (Fig. 2A). In 19 out of 20 infants, IgA results turned negative on the next sample obtained within 1 month, which was consistent with transmitted maternal IgA (Fig. 2B). Of note, 5 out of these 20 non-infected infants with positive IgA had a comparative WB IgM profile identical to their mother, suggesting contamination with maternal blood. One infant without congenital toxoplasmosis had another positive ISAGA IgA (index = 6) during the checkup at 4 months, while it was negative at D3. From the 20 uninfected infants with at least one positive IgA during follow-up, 73 sera were collected, of which 28 were positive. Overall, of 28 positive sera observed in uninfected infants, 26 probably resulted from maternal transfer, while 2 were late false-positive results of unknown origin. The follow-up of those 20 infants showed a clearance of maternal IgG, normal imaging, and absence of clinical signs.

Among the 40 infected babies with positive IgA during serological follow-up, 35 (87.5%) were positive within 1 month after birth (Fig. 2A). The mean time to detect positive IgA in infected neonates was 7.7 ± 13 days. In 18 out of 40 (45%) infants, subsequent samples were also positive, and the mean duration until clearance was higher than for uninfected infants (Fig. 2B). Within the first 10 days of life, 32 out of 56 congenitally infected infants had at least one serum with positive IgA (ISAGA ≥6 or Platelia ≥1); thus, the sensitivity for early neonatal diagnosis was 57.1%. The overall sensitivity and specificity were 71.4% and 97.3%, respectively. The negative predictive value and positive predictive value (PPV) were 94.3% and 69.8%, respectively (Table 1). When doubtful results were considered as positive, i.e., ISAGA ≥3.0 or Platelia ≥0.8, there was only a slight gain in sensitivity (78.6% vs 71.4%), while the PPV decreased dramatically (50.0% vs 69.8%) (Table 1). Indeed, 45 and 8 doubtful results were obtained in uninfected and infected neonates, respectively (Table 2). These doubtful results were more likely to be observed during the first 10 days of life (83%). The sensitivity of IgA testing was higher before than after 10 days of life (63.0% vs 42.2%), whereas the positive predictive value increased when IgA was tested after 10 days of life (90.5% vs 63.9%) (Table 2).

**TABLE 1:** Performance of anti-*Toxoplasma* IgA assay for the diagnosis of congenital toxoplasmosis^*a*^^,^^*b*^

	Sensitivity % (*n*/*N*)	Specificity % (*n*/*N*)	PPV % (*n*/*N*)	NPV % (*n*/*N*)
IgA positive if ISAGA ≥6 or Platelia ≥1	71.4 (40/56)	97.3 (1,017/1,045)	69.8 (65/93)	94.3 (1,017/1,078)
IgA positive if ISAGA ≥3 or Platelia ≥ 0.8	78.6 (44/56)	93.0 (972/1,045)	50.0 (73/146)	94.8 (972/1,025)

^*a*^ NPV, negative predictive value; PPV, positive predictive value.

^*b*^ Specificity, PPV, and NPV were calculated using all included sera. Sensitivity was calculated based on the first positive sample of congenitally infected neonates.

**TABLE 2:** Results of anti-*Toxoplasma* IgA according to age at blood sampling

Variable^*a*^	Patient data at the age of sampling		
	<10 days	≥10 days	All ages
No. of doubtful IgA with no CT ^b*b*^*^,^*^*c*^	39	6	45
No. of samples	632	539	1,171
No. of sera from children with CT	78	48	126
No. of sera from children with no evidence of CT	554	491	1,045
No. of true positives	46	19	65
No. of false positives	26	2	28
No. of doubtful IgA with CT ^*b*^	5	3	8
No. of true-negative results	489	483	972
No. of false-negative results	27	26	53
Sensitivity (%)	63.0	42.2	55.1
Specificity (%)	95.0	99.6	97.2
Positive predictive value (%)	63.9	90.5	69.9
Negative predictive value (%)	94.8	94.9	94.8

^*a*^ Doubtful IgA : ISAGA scores ranging from 3 to 5; Platelia IgA index ranging from 0.800 to 0.999.

^*b*^ Not included for sensitivity, specificity, PPV, and NPV calculation.

^*c*^ CT, congenital toxoplasmosis.

### Clinical value of anti-*Toxoplasma* IgA for the diagnosis of congenital toxoplasmosis

**Fig 3:**
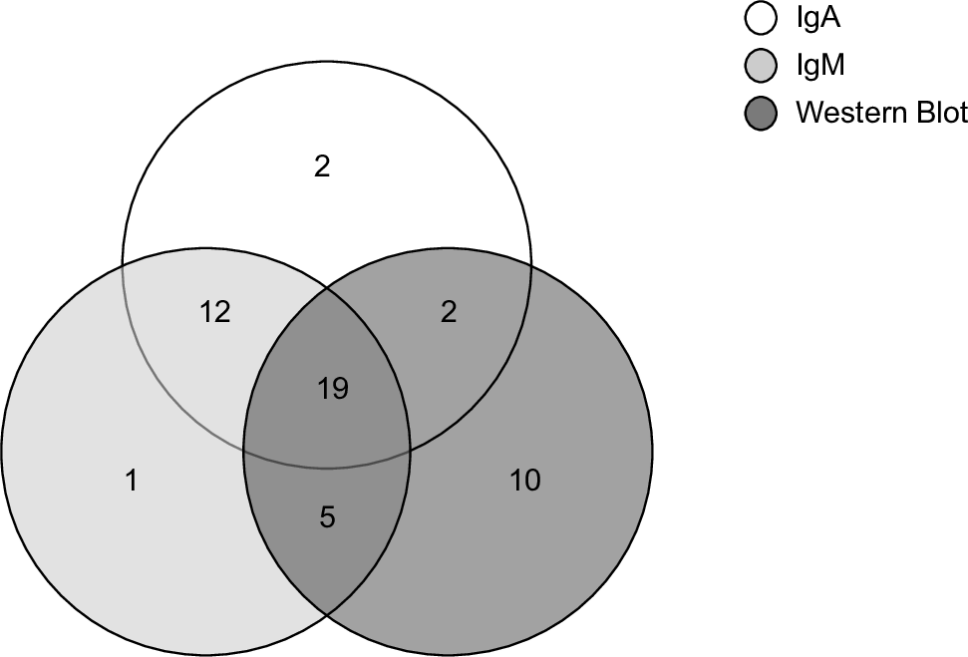
Venn diagram of the earliest positive serological test or combination of tests in congenitally infected infants (*n* = 51). WB: comparative mother/neonate Western blot.

Of the 56 infants diagnosed with congenital toxoplasmosis, 24 (43%) did not benefit from prenatal diagnosis because *Toxoplasma* seroconversion was diagnosed late during the third trimester, underlining the importance of serological follow-up of all infants. *Toxoplasma* PCR was performed on 44 placentas at birth, of which 77% (*n* = 34) were positive. In five cases of congenital toxoplasmosis, no molecular screening was performed, whether on prenatal amniotic fluid or at delivery on placenta, because of a very late maternal seroconversion during pregnancy (33, 36, and 40 weeks or peripartum). In the 40 infected infants with at least one positive IgA, anti-*Toxoplasma* IgM, neosynthesized IgG or IgM on comparative mother/newborn WB, both IgM and neosynthesized IgG or IgM on WB were simultaneously positive in 12 cases (30.0%), 2 cases (5%), and 19 cases (47.5%), respectively. Anti-*Toxoplasma* IgA was the earliest positive serological test, suggestive of congenital infection, in 2 newborns (5%) (Table 2, Fig. 3). In one of those cases, ISAGA IgA index was 10 at birth and turned negative at 1 month in the absence of specific treatment. In the second case, IgA was only moderately positive (Toxo ISAGA IgA = 7 at birth) and presented with a decreasing index at D3 (ISAGA IgA index = 5; i.e., doubtful). Comparative WB at birth concluded with a maternal contamination of the cord blood because of the similar IgM profiles, thus IgA result was overlooked. Of the 16 infants with no IgA detection after birth, 5 had a positive WB, and 5 had both positive IgM and WB. The six remaining infants never had any positive serological test (ISAGA IgA or IgM and WB) during follow-up and were diagnosed via PCR on amniotic fluid or placenta or persistent IgG (Table 3). Of the nine infants with positive WB, three had neosynthesized IgG and IgM; three had IgM alone; and four had IgG alone (Table 3, Fig. 3).

**TABLE 3:** Comparison of post-natal diagnostic tests in the congenitally infected infants according to the trimester of maternal infection^*f*^

Diagnostic criteria	Number (%) of infected infants fulfilling the diagnostic criteria according to the trimester of maternal infection		
	First trimester (*n* = 2)	Second trimester (*n* = 21)	Third trimester (*n* = 29)
IgA detection alone	1 (50)^*a*^	1 (4.8)^*a**,**b*^	0
IgA + IgM	0	3 (14.3)^*d*^	9 (31.3)^*d*^
IgA + WB	0	1 (4.8)^*e*^	0
IgA + IgM + WB	1 (50)^*e*^	2 (9.5)^*d*^	13 (44.8)^*d*^
IgM detection alone	0	0	1 (3.4)^*e*^
WB detection alone	0	7 (33.3)^*d*^	1 (3.4)^*e*^
IgM + WB	0	1 (4.8)^*e*^	5 (17.2)^*d*^
Long-term IgG	0	5 (23.8)^*c**,**d*^	0
Positive placenta PCR	1/1 (100)	13/19 (68.4)	17/21 (80.9)

^*a*^ IgA at 1 month was negative.

^*b*^ Not taken into account because of a maternal contamination of the cord blood at birth.

^*c*^ Of which four had a PCR-positive prenatal amniotic fluid.

^*d*^ Some had a positive quantitative PCR (qPCR) result on placental tissue.

^*e*^ These patients also had a positive qPCR result on placental tissue.

^*f*^ Four infants are not represented in this data because of an unknown trimester of maternal infection.

A more frequent detection of specific IgA was observed in cases where maternal seroconversion occurred in late pregnancy. In fact, 90% (*n* = 26/29) of children born from mothers who seroconverted during the third trimester had at least one IgA-positive serum during follow-up. Those born from mothers who seroconverted during the second semester presented with positive IgA in less than 40% (*n* = 8/21) of cases (Table 4). There were no significant differences in anti-*Toxoplasma* IgA positivity between positive and negative amniotic fluid PCR results. An IgA-positive serum was found in 13 (54.2%) out of 24 and in 4 out of 8 (50%) infants with positive and negative prenatal diagnoses, respectively.

**TABLE 4:** Frequency of anti-*Toxoplasma* IgA detection in infants with congenital toxoplasmosis, depending on the trimester of maternal infection during pregnancy

Trimester of maternal infection	Frequency of IgA positivity, *n*/*N* (%)	Mean time to detect IgA (days)
First	2/2 (100)	0 ± 0
Second	8/21 (38)	1.3 ± 1.4
Third	26/29 (90)	10.9 ± 15.5
Unknown	4/4 (100)	2.0 ± 1.8

## DISCUSSION

Post-natal serological follow-up is primarily used to detect infected neonates who were not diagnosed by prenatal diagnosis. Indeed, amniotic fluid analysis is rarely performed when the seroconversion occurs too late during the pregnancy, as observed in 43% of cases in our study, or when infection is highly suspected at the beginning of pregnancy but not proven. Parasite DNA detection in the placenta also has a high positive predictive value and a good sensitivity for congenital infection (8) but is not sufficient alone to make the diagnosis, while PCR on cord blood has a poor sensitivity. Serological follow-up starting at birth is therefore essential to rule out congenital toxoplasmosis in cases where prenatal diagnosis was not performed or inconclusive. Testing for anti-*Toxoplasma* IgA was added in the serological workup in the 1990s (13, 14), and various studies showed that it improved the sensitivity of neonatal diagnosis when combined with IgM detection (15–17). However, those studies were performed at a time when recommendations for management of pregnancies with *Toxoplasma* infection were less formalized and not universally applied. For example, the delay in mother treatment and the treatment scheme could have positively influenced the performance of IgA results. Besides, prenatal diagnosis was less efficient (16, 18). Molecular diagnosis has now been improved by the use of quantitative PCR methods targeting *rep529* (19, 20), reaching a sensitivity and specificity of about 90% and 100%, respectively (21, 22), and allowing prompt treatment of infected fetuses. This effectiveness in patient care could contribute to a decrease in sensitivity of diagnostic markers, as recent studies have shown that maternal treatment during pregnancy reduced the sensitivity of IgM serological assays used to diagnose congenital toxoplasmosis in the neonate (12). An impact of maternal treatment on the positivity of PCR results at birth in the placenta has also been shown (8).

The discontinuation of the Toxo-ISAGA IgA by BioMérieux and, more recently, the withdrawal of Platelia Toxo IgA, two reference assays for IgA detection, led us to re-assess the value of IgA detection in the framework of neonatal diagnosis. Overall, the sensitivity of IgA detection at birth (<5 days of life) was similar to that reported by Bessières et al. (17) or by Lévêque et al. (23), who both reported a sensitivity of 60% using an ELISA technique. Our results were better than those found by Murat et al. (24), who showed a sensitivity of ISAGA of only 47.8% (*n* = 11/23) and 52.9% (*n* = 9/17) on the first neonatal serum or cord blood, respectively, albeit on a small series. Interestingly, an older study by Pinon et al. (25) had shown a sensitivity of ISAGA IgA which reached 72%, but the threshold of positivity was not indicated.

In the recent study by Lévêque et al. (23), the sensitivity of IgA at birth was lower in infants born to treated mothers than in those from untreated mothers (58.4% vs 68.7%, respectively), although the difference was not statistically significant (*P* = 0.06). The difference in IgA detection observed in the study by Guegan et al. (12) between newborns from treated and untreated mothers was not significant either (68.4% vs 75.5%, respectively), while it was highly significant for IgM detection by ISAGA or WB. This peculiarity could be an advantage, but it is counterbalanced by a poorer positive predictive value, as seen in the present study. These positive results need to be checked on a second sample, which usually shows a rapid clearance of IgA in the absence of congenital infection. Unlike IgM, there is no commercialized WB to compare mother and newborn IgA; thus, there is no simple way to bring out cord blood contamination. In our series, all positive samples from uninfected infants were negative at 5 days of life, except two, which tested positive at 42 and 132 days (Fig. 2B), maybe due to the difficulty of appreciation of the agglutination score of ISAGA with the naked eye, which is subjective. The specificity of IgA for the diagnosis of congenital toxoplasmosis in our study (93.0% or 97.3%, depending on the threshold of positivity) was similar to those reported by Murat et al. with values of 97.3% (*n* = 109/112) and 97.4% (*n* = 75/77) on neonatal serum and cord blood, respectively (21), as well as to other studies (23, 26), but it might depend on the number of uninfected babies included. It does not seem to depend on the IgA technique used (ELISA or ISAGA), as various techniques were used in the above-cited studies. Interestingly, it appears that it is poorly related to contamination of cord blood with maternal blood and rather suggests transfer of low levels of maternal IgA *in utero*, as described previously (27).

In our data set, IgA was generally not detected earlier than other serological parameters indicative of congenital toxoplasmosis, except in two cases. In those two cases, IgA decreased on subsequent samples, suggesting initial contamination by maternal blood, which was actually proven for one case through identical IgM profile on comparative WB. In the other case, WB was normal (transmitted IgG) and IgM was not detected by ELISA nor WB. Anti-*Toxoplasma* IgA was more frequently positive at birth when maternal infection occurred on the third trimester of pregnancy, but in that situation, IgM was always simultaneously positive (100% cases). The mean time to obtain a positive IgA result appeared higher (not significant) when infection was acquired during the third trimester, in relation to few cases of very late maternal infection diagnosed at delivery, on the basis of a positive IgM detection, confirmed by an IgG increase 1 or 2 weeks later. Overall, this poor added value of IgA is in agreement with the study by Denis et al. (28), who showed that IgA was the only positive test in only 2% of infected infants and was generally the test with the lowest sensitivity, whatever the time of sampling. Similarly, in our experience, IgA did not provide a faster diagnosis than IgM or comparative Western blotting, and in the two cases where it was the sole positive test, it was overlooked because of its poor PPV at birth. Overall, our study suggests that anti-*Toxoplasma* IgA is not a discriminatory serological parameter for the diagnosis of congenital toxoplasmosis.

Our study has some limitations, as it was conducted in a country where prenatal serological follow-up of pregnant women is the rule, allowing rapid treatment in case of primary infection, which can contribute to lowering the antibody response in the fetus and the neonate. The rate of IgA detection could probably be higher in newborns from countries where maternal screening and treatment are not implemented. Indeed, the positivity rate observed in the study by Olariu et al. in the United States (29) was higher (80.4%) than in any recent European study, and IgM detection was even higher (92.4%). In conclusion, our results showed that the diagnosis of congenital toxoplasmosis should not be affected by the lack of those two reference assays for the detection of anti-*Toxoplasma* IgA, since the remaining serological tools available to date, i.e., IgM assays of high sensitivity (11) and comparative WB, outperformed IgA detection in terms of earliness, specificity, and sensitivity for post-natal diagnosis. To our knowledge, no other commercialized assays certified for *in vitro* diagnosis are available in France nowadays. Two studies reported the use of ELISA IgA assays but did not evaluate them (30, 31). Given the recent change in the availability of serological assays among countries, the recommendations for the diagnosis of congenital toxoplasmosis should be actualized (32).

## Data Availability

Results and anonymized data are available on the following zenodo repository, accessible at https://doi.org/10.5281/zenodo.15490613
